# CDK4/6 and aromatase inhibitors as first‐line treatment in metastatic high‐grade neuroendocrine carcinoma of the breast: A case report

**DOI:** 10.1002/ccr3.9180

**Published:** 2024-07-16

**Authors:** Dionysia N. Zouki, Vasiliki‐Elpida Kardara, Stephanie Ioannou, Eleni Arvanitou, Konstantinos Exarchos, Konstantinos Gkikas, Stefanos Konstantoudakis, Sophocles Lanitis, Stylianos Benakis, Dimitrios Tryfonopoulos

**Affiliations:** ^1^ Second Department of Medical Oncology Agios Savvas Cancer Hospital Athens Greece; ^2^ Department of Pathology Korgialenio—Benakio Hellenic Red Cross Hospital Athens Greece; ^3^ Unit of Surgical Oncology, Second Surgical Department Korgialenio—Benakio Hellenic Red Cross Hospital Athens Greece; ^4^ Radiology Department Evangelismos Hospital Athens Greece

**Keywords:** breast neuroendocrine carcinoma, CDK4/6 inhibitors, hormonal therapy, treatment strategy

## Abstract

**Key Clinical Message:**

There is no consensus regarding the therapeutic approach of breast neuroendocrine carcinomas (NECs). As most NECs are hormone receptor positive and HER‐2 negative, we suggest that endocrine‐based strategies may play a leading role. Here, we report a new treatment strategy by incorporating CDK4/6 inhibitors in the therapeutic armamentarium.

**Abstract:**

Primary neuroendocrine neoplasms of the breast constitute a rare entity. They are characterized by predominant neuroendocrine differentiation and are further divided into well‐differentiated neuroendocrine tumors and poorly differentiated (high‐grade) neuroendocrine carcinomas (NECs). Regarding their therapeutic approach, there are no standardized guidelines. Herein, we present the first case ever reported, concerning a female patient with de novo metastatic breast NEC who received hormonal therapy, a combination of a CDK4/6 inhibitor palbociclib with letrozole and triptorelin, as first‐line treatment with significant clinical and radiological response. As most NECs are estrogen receptor and/or progesterone receptor positive and HER‐2 negative, we suggest that hormonal therapy may play a leading role even in the first‐line setting. The present report provides a new treatment strategy by incorporating CDK4/6 inhibitors in the therapeutic armamentarium of breast NECs.

## 1 INTRODUCTION

Primary neuroendocrine neoplasms (NENs) of the breast constitute a rare entity which is not clinically distinct from other more common tumor types. According to the latest WHO classification,^1^ the term NEN encompasses all neoplasms with predominant neuroendocrine (NE) differentiation (presence of histologic NE features in more than 90% of the tumor), by further dividing them to well‐differentiated neuroendocrine tumors (NETs) and poorly differentiated (high‐grade) neuroendocrine carcinomas (NECs).^2^ Both entities express diffuse NE markers, such as synaptophysin and chromogranin. In NENs of the breast, the number of mitoses is the main parameter that affects the grading system. NECs are further subdivided to small cell and large cell NECs, both accounting for approximately 0.1% of all breast cancers, and unlike other NETs no clinical syndromes related to hormone production are being documented. These carcinomas tend to have a more aggressive behavior and are being linked to poor prognosis. By the time of diagnosis 19%–30% of NECs have already distant metastatic spread. Most NECs are hormone receptor positive and HER‐2 negative, adopting a luminal like phenotype.^1^, ^2^, ^3^

Most of the data concerning breast NECs originate from case reports and small retrospective series.^4^, ^5^ There are no standardized guidelines regarding their therapeutic approach. For early staged disease, surgery is the mainstay of treatment followed by chemotherapy, endocrine therapy and targeted therapy according to the receptor status. In the metastatic setting, there is a sufficient lack of evidence concerning the most appropriate chemotherapy regimen. In general, NECs are being treated with platinum/etoposide chemotherapy protocols.^2^

As previously noted, most NECs are estrogen receptor (ER) and/or progesterone receptor (PR) positive, indicating that hormonal therapy may play a leading role. To the best of our knowledge, this is the first case reported regarding a female patient with de novo metastatic breast NEC who received hormonal therapy as first‐line treatment with significant clinical and radiological response.

## 2 CASE HISTORY

A 46‐year‐old nulliparous premenopausal woman without any significant medical, surgical or family history, presented to our oncology clinic with a palpable breast mass in the upper outer quadrant of her left breast. On clinical examination, there was no cervical or supraclavicular adenopathy. Staging with computed tomography scans revealed a 30 mm mass in the left breast, left axillary lymphadenopathy, as well as multiple metastatic lesions in the liver and the bones.

## 3 METHODS

**FIGURE 1:**
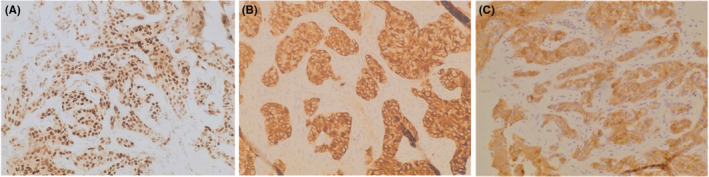
On immunohistochemistry, tumor cells show diffuse positive staining for estrogen receptor (A), chromogranin (B), and synaptophysin (C) (×200).

The patient underwent a core needle biopsy of the breast mass, which was consistent with high‐grade NE breast carcinoma, ER (100%) (Figure 1A) and PR (50%) positive and HER‐2 negative (0). Further analysis revealed Ki67 index of 60% and positivity for chromogranin and synaptophysin (Figure 1B,C). By the time of diagnosis, she was mildly symptomatic, mainly due to bone metastasis‐associated pain. In conclusion, it was a stage IV NEC of the breast, luminal B‐like, HER‐2 negative.

The case was discussed in the multidisciplinary tumor board (MTB) and considering that chemotherapy is the main treatment of patients with metastatic NECs, adapting regimens that have historically been used for other NECs, it was decided to proceed with the combination of carboplatin and etoposide.

However, when the treatment plan was announced to the patient, she clearly refused chemotherapy being afraid and extremely anxious of potential side effects. Since the tumor presented a luminal phenotype and considering the proven benefit from using CDK4/6 inhibitors in hormone receptor‐positive and HER‐2‐negative metastatic breast cancer, the MTB proposed as an alternative option, hormonal therapy, an oral combination of a CDK4/6 inhibitor palbociclib (125 mg once daily, 3 weeks on and 1 week off) with 2.5 mg letrozole once daily and 3.75 mg triptorelin as intramuscular injection every 4 weeks.

## 4 CONCLUSION AND RESULTS

**FIGURE 2:**
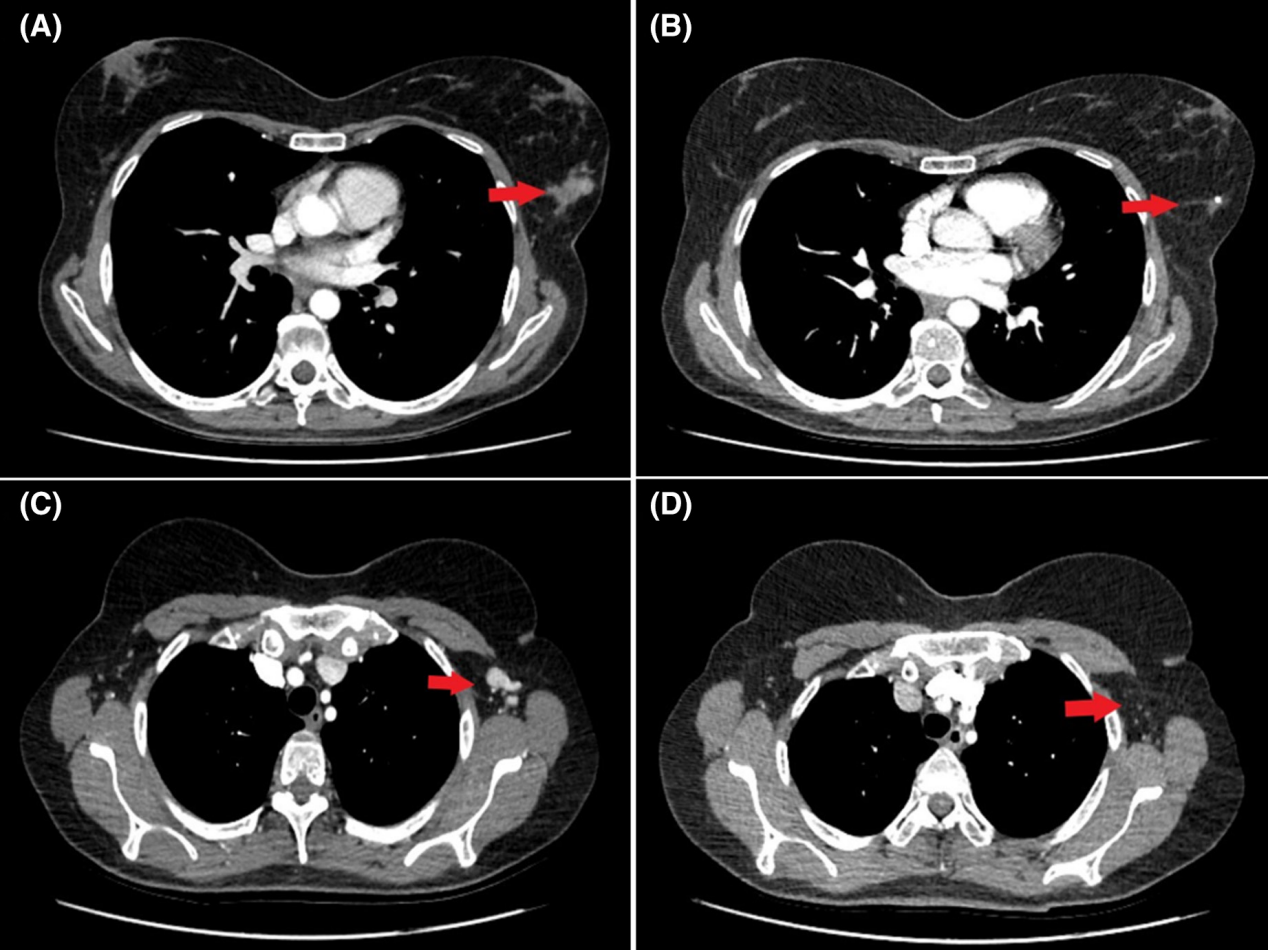
Contrast‐enhanced computed tomography scans of the thorax. Left breast mass before treatment initiation (A) and after 3 months of therapy (B). Left axillary lymphadenopathy before treatment initiation (C) and 3 months after (D). Red arrows indicate the tumor area.

**FIGURE 3:**
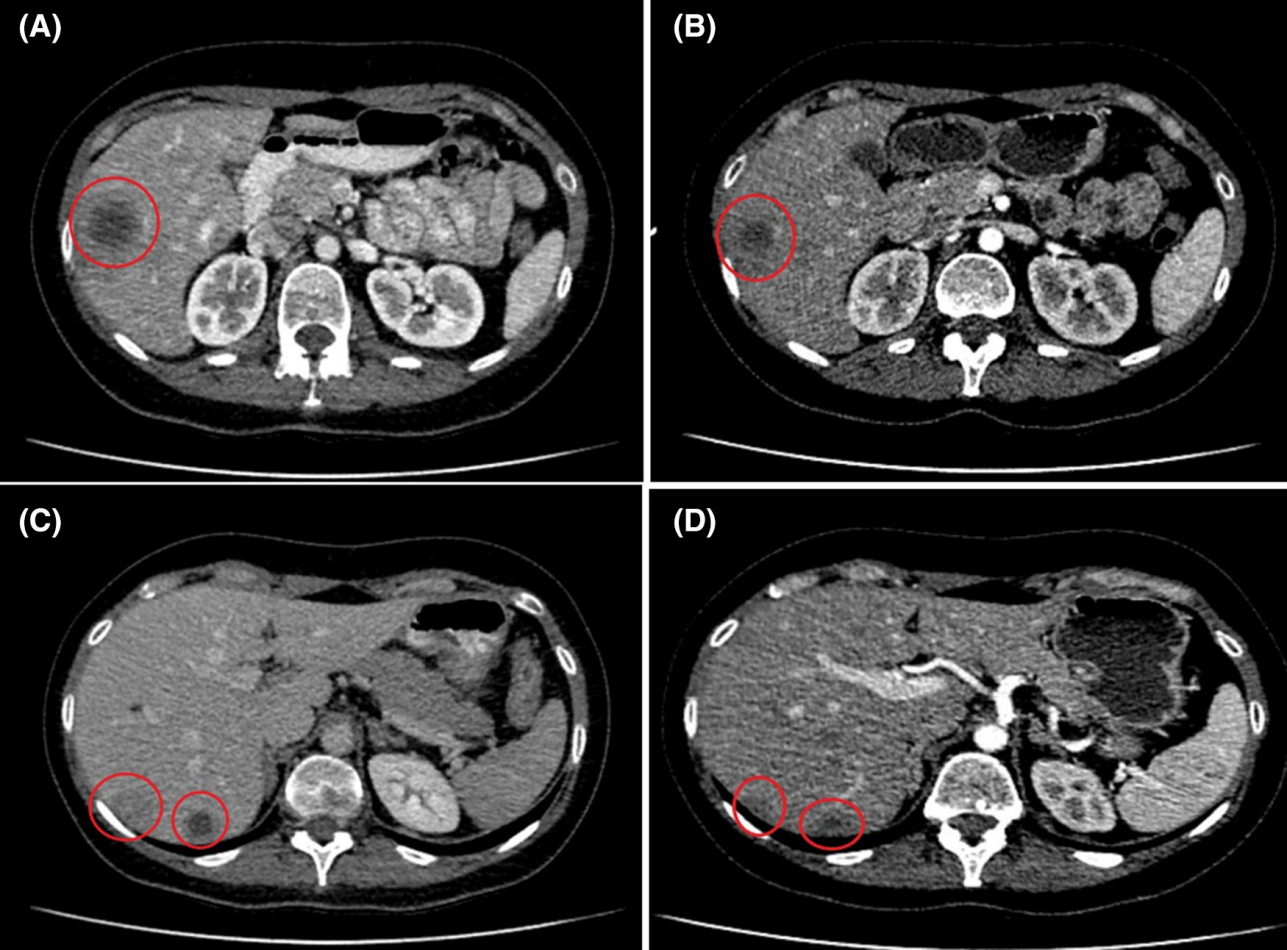
Contrast‐enhanced computed tomography scans of the abdomen showing the metastatic liver lesions before treatment initiation (A, C) and 3 months after (B, D). Red circles highlight the tumor areas.

The patient tolerated this regimen well with immediate clinical improvement within the first 2 weeks. She had no need for analgesics, she was no longer symptomatic and expressed her willingness to start working again. Computed tomography scans after 3 months indicated complete response in the breast mass (Figure 2A,B) and the axillary lymph nodes (Figure 2C,D), as well as significant tumor shrinkage in most metastatic liver lesions (Figure 3). After 10 months of treatment, she continues to be progression free, maintaining a very good partial response to her palbociclib‐based therapy.

## 5 DISCUSSION

Primary NE breast carcinomas are very uncommon. They are characterized by high‐grade NE morphology along with the presence of neurosecretory granules and a diffuse immunoreactivity of NE markers.^1^ Due to their rarity, the standard treatment strategy remains controversial. It appears that these patients should be treated in a similar way to invasive breast cancer according to the size and the stage of the disease.^6^

Since metastatic and primary NE breast carcinomas require different therapeutic approaches, differential diagnosis between these two entities is essential. The presence of an intraductal component with the same pathologic features is highly suggestive of breast origin. Immunohistochemistry also plays a crucial role, with the most specific markers indicative of primary breast tumor to be mammoglobin, GATA 3 and GCDFP15 (gross cystic disease fluid protein)^7^, ^8^ as well as ER and PR positivity.^3^

Breast NECs are usually HER‐2 negative and hormone receptor positive, adopting a luminal A like phenotype. Only two cases in the literature are reported as HER‐2 positive,^8^, ^9^ making this an extremely rare condition. Based on data mainly originating from case reports and series,^4^, ^5^ high‐grade metastatic NECs are being treated with chemotherapy regimens used for NEC of the lung and other similar high‐grade poorly differentiated NETs.^3^, ^6^ However, taking into account the luminal phenotype, endocrine‐based strategies have been recently examined in different settings of the disease. Do Hyung Lee et al. presented the first patient with primary breast NEC with clinical features of inflammatory carcinoma, who did not respond neither to cisplatin based systemic treatment nor to tamoxifen.^10^ Moreover, few years later, Shanks A et al. reported a case of a female patient with metastatic breast NEC with progressive disease after first‐line treatment with cisplatin–etoposide and maintenance tamoxifen but with substantial response to the combination of palbociclib and fulvestrant.^11^ More recently, Guerra et al. reported an interesting case of a premenopausal woman with oligometastatic breast NEC, who received weekly paclitaxel and achieved complete response in all metastatic sites. Given the radical aim of treatment within the context of oligometastasis and the patient's will, she underwent radical right mastectomy. Since the tumor was hormone receptor positive and HER‐2 negative, she was offered, as adjuvant‐like treatment, a combination of CDK4/6 inhibitor with endocrine therapy, tolerating the treatment well and being disease‐free in her last follow‐up.^12^ Although our current knowledge regarding therapeutic management of breast NECs is scarce and based mainly on case reports, CDK4/6 inhibitors are being proposed as another potential targeted therapy.^13^ Despite the limitations originating from the lack of clinical trials, we must acknowledge that endocrine based treatment has a crucial role in hormone receptor positive breast cancers, including NECs.

Herein, we report for the first time in literature, the case of a patient with de novo metastatic breast NEC, who achieved a remarkable response to first‐line treatment with palbociclib and letrozole. During the last years, CDK4/6 inhibitors have changed the treatment landscape of hormone receptor‐positive and HER‐2‐negative breast tumors offering large progression‐free intervals to many patients with invasive breast cancer. As far as, endocrine‐based therapy is concerned, Zhang et al. presented a case of a 28‐year‐old female patient with breast NEC, who received 3 months of neoadjuvant letrozole and goserelin, achieving significant tumor reduction by almost 78% according to response evaluation criteria in solid tumors 1.1 (RECIST criteria version 1.1).^14^ Taking into consideration the luminal phenotype in the majority of breast NECs, hormonal treatment may be the mainstay in the therapeutic armamentarium across all stages of the disease.

By further exploring the more recent molecular insights, it has been reported that breast NECs harbor PIK3CA mutations in a varying percentage (7%–33%), which is less frequent than the other more common breast tumor types. As a result, targeting PIK3CA may be a treatment option in metastatic NECs, given the evidence of alpelisib use in hormone receptor‐positive and HER‐2‐negative breast cancer.^15^, ^16^ Moreover, TROP‐2 protein expression has also been documented in a proportion of breast NECs, indicating that antibody drug conjugates, such as sacituzumab govitecan, may prove useful in therapeutic management.^2^, ^3^

## 6 CONCLUSIONS

Breast NECs represent an extremely rare and heterogenic entity. Due to limited scope of knowledge regarding their biologic behavior, there is no consensus for their therapeutic approach. Some patients prove to be resistant to conventional chemotherapy and endocrine‐based strategies seem to gain significant attention during the last years, including CDK4/6 inhibitors. Better understanding of the underlying mechanisms and further research is warranted in order to be able to set a more tailored treatment plan.

## AUTHOR CONTRIBUTIONS

**Dionysia N. Zouki:** Conceptualization; data curation; writing – original draft; writing – review and editing. **Vasiliki‐Elpida Kardara:** Data curation; supervision. **Stephanie Ioannou:** Data curation; validation. **Eleni Arvaniti:** Writing – original draft; writing – review and editing. **Konstantinos Exarchos:** Investigation; methodology. **Konstantinos Gkikas:** Investigation; methodology. **Stefanos Konstantoudakis:** Resources; software; visualization. **Sophocles Lanitis:** Data curation; resources; validation. **Stylianos Benakis:** Visualization; writing – review and editing. **Dimitrios Tryfonopoulos:** Conceptualization; project administration; writing – original draft; writing – review and editing.

## FUNDING INFORMATION

No funding was received to assist with the preparation of this manuscript.

## CONFLICT OF INTEREST STATEMENT

The authors declare no conflict of interest.

### ETHICS STATEMENT

Ethics approval by committee was not required for this case report. It was conducted in accordance with the 1964 Helsinki Declaration and its later amendments or comparable ethical standards.

### CONSENT

Written informed consent was obtained from the patient to publish this report in accordance with the journal's patient consent policy.

## Data Availability

The datasets used and/or analyzed during this study are included in this paper and shall be available from the corresponding author upon request.
